# High Prevalence of 5T4/Trophoblast Glycoprotein in Soft Tissue Sarcomas

**DOI:** 10.3390/cancers13194841

**Published:** 2021-09-28

**Authors:** Patrick Groothuis, Nicola Penel, Antoine Italiano, Nuria Kotecki, Fred Dijcks, Wim Dokter

**Affiliations:** 1 Byondis B.V., Microweg 22, 6503 GB Nijmegen, The Netherlands; fred.dijcks@byondis.com (F.D.); wim.dokter@byondis.com (W.D.); 2 Medical Oncology Department, Centre Oscar Lambret, 59000 Lille, France; n-penel@o-lambret.fr; 3 Medical Oncology Department, Institut Bergonié, 33000 Bordeaux, France; A.Italiano@bordeaux.unicancer.fr; 4 Medical Oncology Department, Institut Jules Bordet, 1000 Brussels, Belgium; nuria.kotecki@bordet.be

**Keywords:** soft tissue sarcoma, 5T4, trophoblast glycoprotein, immunohistochemistry, cell surface antigen

## Abstract

**Simple Summary:**

Soft tissue sarcoma (STS) is a heterogeneous group of hard-to-treat malignancies from mesenchymal or connective tissues. The treatment options in case the cancer relapses after surgery or is at an advanced state are limited, and in all cases the response rates are low. Targeted therapy with antibody drug conjugates may provide an alternative. To this end, molecules that are specifically (over)expressed on the surface of the tumor cells are needed. In this study, we show that 5T4/trophoblast glycoprotein, a molecule with limited expression in healthy tissues, is prominently expressed on the cell surface of many STS subtypes and can be used for such an approach.

**Abstract:**

The expression of 5T4/trophoblast glycoprotein was evaluated in several histological subtypes of soft tissue sarcoma (STS) to determine whether the prevalence and level of expression of this membrane-associated glycoprotein is sufficient for use in targeted therapies. Tumor tissue microarrays containing cores from different histological subtypes of STS were stained using a standardized immunohistochemical staining method to detect 5T4; the level of staining was assessed using a semi-quantitative scoring method. No 5T4 staining was seen in the angiosarcomas and liposarcomas investigated in this study. 5T4 staining in the other STS subtypes was seen in more than 50% of cases, warranting further investigation into whether this antigen could evoke an anti-tumor immune response or can be used as target for the delivery of more potent toxins through antibody drug conjugates.

## 1. Introduction

Soft tissue sarcomas (STS) comprise a heterogeneous group of hard-to-treat malignancies originating from mesenchymal or connective tissues. They account for about 1% of cancers in adults [1] and 15% of cancers in children. In patients under 20 years old, STS is among the top five causes of cancer deaths in the United States [2,3]. The high incidence in children is driven by the occurrence of rhabdomyosarcomas [3], which often develop in the head and neck region [4]. The 5-year survival of STS on average is about 65% but ranges from 15% to about 80% depending on the tumor type, tumor grade at diagnosis, whether the cancer has metastasized, and whether a patient underwent surgery or not [5,6]. In about 20–25% of confirmed STS cases, the disease has already metastasized at presentation [5].

Soft tissue sarcomas can only be diagnosed by careful microscopic examination of tissue collected through core-needle or incisional biopsy. STSs are then classified according to the presumed tissue of origin, immunohistochemical markers, and genetic rearrangements into one of more than 70 different subtypes. The complexity of differential diagnosis is further illustrated by the fact that about 10–15% of STS cases remain undefined [7]. Subsequent staging using imaging techniques is important to determine the most effective course of treatment and surgical decision making.

For localized disease, the cornerstone of treatment is based on surgery with or without peri-operative radiotherapy/chemotherapy. The main objective is to minimize the risk of local and systemic recurrence and render the least functional impairment.

In case of advanced or metastasized disease systemic treatments are recommended. The mainstay of systemic therapy for metastatic STS have been doxorubicin and ifosfamide, and lately other drugs like gemcitabine, trabectedin, eribulin, pazopanib, dacarbazine, and taxanes have also shown clinical activity, but the response rates are disappointingly low.

Molecular profiling has confirmed that there is an enormous diversity between and within the STS subtypes, and given this diversity and complexity, clinical management of STS is quite challenging. However, these studies sometimes also revealed molecular abnormalities that have led to breakthrough targeted therapies: for instance, the use of imatinib in gastro-intestinal stromal tumors (GISTs) with gain-of-function mutations in the *KIT* gene [8,9]; crizotinib in advanced, inoperable inflammatory myofibroblastic tumors with disease-driving ALK fusions [10]; larotrectinib for the treatment of neurotrophic TRK gene fusions [11]; and the recently approved avapritinib in patients with GISTs driven by a PDGFRA exon 18 mutation [12].

Unfortunately, these targetable genetic alterations are quite rare, whereas for the majority of patients with advanced STS, prognosis is dismal and better therapeutic options need to be found. Individualized targeted treatments have shown to result in better disease control, however, only in small subsets of patients. Alternatively, targeted therapies aimed at common features of STS could benefit larger groups of patients. Despite the mediocre clinical efficacy, preclinical studies have shown encouraging results for different classes of chemotherapeutic agents, like the DNA-damaging agents (i.e., doxorubicin, ifosfamide, dacarbazine, and trabectedin), microtubule polymerization inhibitors (i.e., vincristine, eribulin, and taxanes), and pan-tyrosine kinase inhibitors (i.e., pazopanib) [13]. However, effectivity of available drugs to treat STS is disappointing, probably also due to dose-limiting toxicities, which block the option to treat at sufficiently high dosages. An efficient method to increase the therapeutic window is by coupling highly potent toxins to a targeting carrier molecule, such as an antibody. Conjugating doxorubicin to an antibody, for example, was shown to increase the therapeutic index in a multiple myeloma mouse xenograft model by at least 50-fold [14].

An important aspect of this approach is the availability of cell surface antigens that are selectively expressed or upregulated by the STS cells. In a recent review, Polito et al. discussed the state-of-the-art development of immunoconjugates for the targeted therapy of sarcomas [15]. The use of immunoconjugates has proven effective in certain hematological and epithelial cancers, but their potential in sarcomas has yet to be demonstrated. The results from preclinical studies showed promise and have led to the initiation of several clinical trials, and in some of the trials STS patients are also being recruited. The immunoconjugates being investigated in STS patients, such as antibody drug conjugates (ADCs), immunotoxins, and radioimmunoconjugates, are directed to a variety of cell surface antigens including endosialin (CD248), EGFR, CSPG4, NCAM, GPNMB, uPARAP, ROR2, CD70, and FZD10 [15].

Byondis developed an anti-5T4 ADC using its proprietary linker-drug platform based on a highly potent synthetic duocarmycin analogue and a cathepsin B-sensitive linker [16]. 5T4, or trophoblast glycoprotein, is a heavily glycosylated membrane-associated protein that is typically highly expressed in the developing placenta [17]. It was identified by Hole and Stern, who recognized that the trophoblast demonstrates some functional properties of neoplastic tissue, such as invasiveness of host tissue and escape from immunological surveillance, and set out to find cell surface molecules with restrictive expression on syncytiotrophoblast and cancer cells [17].

5T4 is an interesting tumor target because in adult tissues the expression of 5T4 is suppressed [18], but in tumor cells, the brake on 5T4 expression is released and 5T4 is upregulated in multiple cancer types, and 5T4 overexpression is often correlated with poor prognosis [19,20]. This is likely related to the fact that 5T4-positive cancer cells often have stem cell-like properties [21] and that 5T4 plays key roles in adhesion, cytoskeletal organization and motility, and the epithelial-to-mesenchymal transition associated with metastasis [22,23].

During embryonal development, 5T4, also referred to as the Wnt-activated inhibitory factor-1 (*Waif1*), is not only involved in neurectoderm patterning, but is also expressed in endomesodermal cells during late blastula stages and is important for the dorsoventral patterning of the mesoderm [24]. It is therefore no surprise that in many solid tumors, the mesenchymal cells or cancer-associated fibroblasts also express 5T4 [18,20], and significant 5T4 expression was found in malignant pleural mesotheliomas [25], which arise from the mesodermally derived mesothelium.

To date, 5T4 expression has been reported in only one single case of STS, a fibrosarcoma, and it was 5T4 positive [18]. In this study, we evaluated the expression of 5T4 in different histological subtypes of soft tissue sarcomas by immunohistochemical staining of tumor tissue microarrays (TMAs), and we confirm that it is widely expressed in STS.

## 2. Materials and Methods

### 2.1. TMAs and Antibodies

Soft tissue sarcoma tissue microarrays (#SO2084, SO2083a, MC805, SO809c, SS1001, and SO2084), were purchased via BioCat GmbH (Heidelberg, Germany). The slides were baked for 2 h at 60 °C prior to storage and shipping. The anti-5T4 rabbit monoclonal antibody (ab134162) and isotype control antibody (ab172730) were ordered from Abcam. OmniMap Rb HRP (cat.nr. 760-4311), ChromoMap DAB kit (cat.nr. 760-159), Hematoxylin (cat.nr. 760-20210), and Bluing Reagent (cat.nr. 760-2037) were purchased from Ventana Medical Systems.

### 2.2. Immunohistochemistry

Immunohistochemical staining (IHC) was performed at Cerba Research/Histalim (Montpellier, France), using the validated staining protocol #332 on the Roche Ventana Discovery XT platform. In brief, sections were deparaffinized and subjected to antigen retrieval for 64 min at 95 °C (CC1 long) in Tris-EDTA buffer pH 7.8. The sections were subsequently incubated for 12 h at room temperature with 2.8 μg/mL of the anti-5T4 antibody (ab134162) or isotype control antibody (ab172730). Antibody binding was visualized using the OmniMap Rb HRP (1:400, 16 min) and the ChromoMap DAB kit (Ventana Medical Systems, Oro Valley, AZ, USA). Sections were counterstained with hematoxylin and bluing reagent. The slides were digitalized with the Hamamatsu Nanozoomer.

### 2.3. Semi-Quantitative Assessment of 5T4 IHC

The immunostaining was evaluated using a semi-quantitative immuno-scoring protocol using the category descriptions listed in Table 1.

## 3. Results

**Figure 1:**
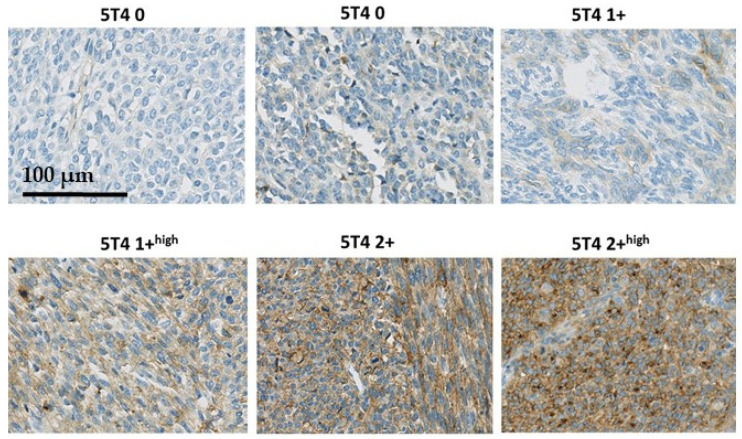
Examples of the 5T4 immunostaining in synovial sarcomas.

**Figure 2:**
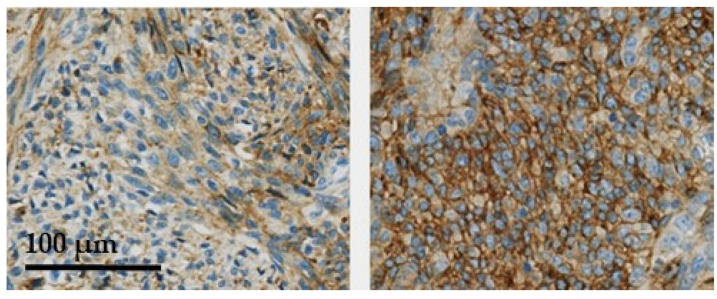
Two synovial sarcoma cases showing clear membrane localization of 5T4 (arrows).

In Figure 1, examples of the 5T4 immunostaining in synovial sarcomas are presented to illustrate how the semi-quantitative immune-scoring method relates to immunostaining seen in the tissues. No STS biopsies were seen with a 3+ score. In Figure 2, two examples are presented of synovial sarcomas showing the clear presence of membrane localization of 5T4.

Core biopsies from 289 STS cases were evaluated, including angiosarcomas (*n* = 12), liposarcomas (*n* = 76), synovial sarcomas (*n* = 88), fibrosarcomas (*n* = 28) dermatofibrosarcomas (*n* = 35), leiomyosarcomas (*n* = 20), rhabdomyosarcomas (*n* = 20), and undifferentiated pleiomorphic sarcomas (*n* = 22). Detailed information regarding age, sex, tumor type, and the location where the STS tissue was collected (when provided), as well as the individual immunoscores, can be found in the Supplementary Materials (Table S1: Patient information and IHC scores). The summary of the immunohistochemical scoring results is presented in Table 2. Angiosarcomas and liposarcomas appear to not express 5T4. In the other histological STS subtypes evaluated, 5T4 membrane staining was observed in more than 50% of the cores (Table 2). More prominent 5T4 expression was found in synovial sarcomas, leiomyosarcomas, and undifferentiated pleiomorphic sarcomas.

## 4. Discussion

Using immunohistochemical staining and a semi-quantitative scoring method, the expression of the glycoprotein 5T4 was evaluated in tissue microarrays containing cores of several histological subtypes of soft tissue sarcoma (STS). Recent studies have shown quite convincingly that, regarding the expression of 5T4, there is a close correlation between 5T4 IHC scores and mRNA expression levels [26,27]. It is therefore plausible to assume that our observations are a true representation of the level of 5T4 expression in the population of STS patients.

5T4 was shown to be prominently expressed in multiple STS subtypes, in which 5T4 membrane staining was observed in 50% or more of the STS cases investigated. The exceptions in this study were angiosarcomas and liposarcomas, which were shown not to express 5T4 in the cores examined. Given the fact that 5T4 expression in normal tissues is limited, these findings advocate for the development of anti-5T4 therapies for the targeted treatment of 5T4-positive STS.

Various therapies targeting cell surface-expressed 5T4 are already under evaluation in the clinic, including vaccines [28], antibody-fusion proteins—such as naptumomab estafenatox [29], bi-specific antibodies [30], and CAR-T therapies [31]—and antibody drug conjugates (ADCs) [16,32]. No 5T4-targeting therapeutic has reached the market yet.

5T4 is not the only placenta-associated protein. Many were found to be overexpressed in cancer; however, none have been described to be expressed in STS sarcoma [33]. Other cell surface antigens that can potentially serve as therapeutic target and that are prominently expressed in STS are NY-ESO-1, EGFR, c-MET, and GD2. The expression of the cancer testis antigen, NY-ESO-1, even though it is highly selective for certain malignancies, is mostly limited to myxoid liposarcomas and synovial sarcomas [34], representing relatively small subsets of STS. Targeted depletion of disialoganglioside/GD2-expressing cells is probably not a desirable approach, as it is expressed by various normal tissues including peripheral sensory nerve fibers, and treatments with the therapeutic antibodies are associated with neuropathic pain [35,36]. EGFR and c-MET are, like 5T4, also widely expressed and activated in STS, and their activated state correlates with a poor prognosis [37,38,39,40,41]. Their value as therapeutic target, however, has yet to be confirmed.

Another key factor related to anti-tumor activity of large molecule formats such as ADCs is tumor accessibility. In other words, are the tumors well vascularized, allowing for good distribution of the therapeutic? Solid tumors often are poorly vascularized because of hypoxia and necrosis, high interstitial fluid pressure or solid stress, and heterogeneous distribution of blood vessels [42,43].

In STS, primary tumors, local recurrences, and distant metastases appear to be well vascularized, sometimes even more so than the surrounding normal tissues [44,45]. Moreover, the distribution of (small) blood vessels in the tumor tissue appears to be quite homogeneous [46,47]. In contrast, little has been reported on the vascularity or vascular density of local recurrent metastases or even pulmonary metastases, even though the lungs are the most common site of distant metastases, accounting for up to 80% of all metastases in most series [48], and pulmonary metastases are the primary cause of death, even after complete resection of the recurrences [44,49]. Early arteriography studies have shown that pulmonary metastases are well vascularized [50,51]. Ratto and coworkers [52] showed, using the isolated lung perfusion procedure, that the distribution of platinum was similar in normal and metastases of soft tissue sarcomas, suggesting that the metastatic tissue is also well vascularized. In fact, to our knowledge, only one study has compared the microvessel density in primary tumors and pulmonary metastases in soft tissue sarcomas [53]. In this study in osteosarcomas, the authors showed that the microvessel density was significantly higher in metastatic than in the primary tumors.

Together, these observations imply that targeted therapeutics can be an effective approach to treat resistant primary STS, recurrent or distant metastases, or might potentially even be used in the neo-adjuvant or adjuvant setting.

## 5. Conclusions

In conclusion, we showed that 5T4 is prominently expressed in various STS subtypes and could potentially serve as an antigen to evoke an anti-tumor immune response or as target for the delivery of toxic payloads through ADCs. The first, in-human dose-finding trial is ongoing (NCT04202705) to evaluate the safety and explore the efficacy of SYD1875, a novel 5T4-targeting ADC based on a cathepsin B-sensitive linker coupled to a highly potent DNA-damaging agent.

## Figures and Tables

**Table 1:** 5T4 immuno-scoring categories.

Score	Description
0	No staining, or 5T4 immunostaining clearly present but no recognizable membrane staining
1+	Mild, recognizable membrane staining in <10% of cells
1+high	Mild membrane staining in ≥10% of cells and moderate membrane staining in <10% of cells
2+	Moderate membrane staining (circumferential or not) in ≥10% of cells
2+high	Moderate membrane staining in ≥10% of cells and strong membrane staining in <10% of cells
3+	Equal to or more than 10% strong (circumferential or not) membrane staining

**Table 2:** Prevalence of 5T4 immunostaining in soft tissue sarcomas.

STS * Subtype	5T4 1+ or Higher	5T4 1+^high^ or Higher
Synovial sarcoma	51/88 (58%)	15/88 (17%)
Fibrosarcoma	17/28 (61%)	3/28 (11%)
Dermatofibrosarcoma protuberans	25/35 (71%)	4/35 (11%)
Leiomyosarcoma	10/20 (50%)	4/20 (20%)
Rhabdomyosarcoma	12/20 (55%)	1/20 (5%)
Undifferentiated pleiomorphic sarcoma	14/22 (64%)	5/22 (23%)
Angiosarcoma	0/12	
Mixed liposarcoma	0/4	
Well-differentiated liposarcoma	0/15	
Myxoid/round cell liposarcoma	0/25	
Conventional liposarcoma	0/26	
Pleiomorphic liposarcoma	0/6	

* STS: soft tissue sarcoma.

## Data Availability

Data is contained within the article and Supplementary Table S1: Patient information and IHC scores.

## Supplementary Materials

The following are available online at https://www.mdpi.com/article/10.3390/cancers13194841/s1, Table S1: Patient information and IHC scores.
